# Genome-wide identification of cyclophilin genes in *Gossypium hirsutum* and functional characterization of a CYP with antifungal activity against *Verticillium dahliae*

**DOI:** 10.1186/s12870-019-1848-1

**Published:** 2019-06-21

**Authors:** Jun Yang, Guoning Wang, Huifeng Ke, Yan Zhang, Lianlian Ji, Lizhi Huang, Chunying Zhang, Xingfen Wang, Zhiying Ma

**Affiliations:** North China Key Laboratory for Crop Germplasm Resources of Education Ministry, Hebei Agricultural University, Baoding, 071001 China

**Keywords:** Cyclophilin, Cotton, Resistance, *Verticillium dahliae*, Antifungal activity

## Abstract

**Background:**

Cyclophilins (CYPs), belonging to the peptidyl prolyl cis/trans isomerase (PPIase) superfamily, play important roles during plant responses to biotic and abiotic stresses.

**Results:**

Here, a total of 79 CYPs were identified in the genome of *Gossypium hirsutum.* Of which, 65 GhCYPs only contained one cyclophilin type PPIase domain, others 14 GhCYPs contain additional domains. A number of *cis*-acting elements related to phytohormone signaling were predicated in the upstream of *GhCYPs* ORF. The expression analysis revealed that *GhCYPs* were induced in response to cold, hot, salt, PEG and *Verticillium dahliae* infection. In addition, the functional importance of *GhCYP-3* in Verticillium wilt resistance was also presented in this study. GhCYP-3 showed both cytoplasmic and nuclear localization. Overexpression of *GhCYP-3* in *Arabidopsis* significantly improved Verticillium wilt resistance of the plants. Recombinant GhCYP-3 displayed PPIase activity and evident inhibitory effects on *V. dahliae in vitro*. Moreover, the extracts from *GhCYP-3* transgenic *Arabidopsis* displayed significantly inhibit activity to conidia germinating and hyphal growth of *V. dahliae*.

**Conclusions:**

Our study identified the family members of cotton CYP genes using bioinformatics tools. Differential expression patterns of *GhCYPs* under various abiotic stress and *V. dahliae* infection conditions provide a comprehensive understanding of the biological functions of candidate genes. Moreover, GhCYP-3 involved in the resistance of cotton to *V. dahliae* infection presumably through antifungal activity.

**Electronic supplementary material:**

The online version of this article (10.1186/s12870-019-1848-1) contains supplementary material, which is available to authorized users.

## Background

The allotetraploid *Gossypium hirsutum* L., the most important fiber crop, is cultivated worldwide because of its high yield [1]. Environmental stresses, such as cold, drought, heat, salinity, various pests and pathogens, threaten cotton growth, yield and fiber quality. For example, an area of about 300 million hectares of cotton is subject to Verticillium wilt and the economic loss is more than RMB 12 billion every year in China. Verticillium wilt, the most serious disease to influence cotton production in China, caused by soil-borne fungus *Verticillium dahliae.* At present, few resistant varieties have been cultivated by traditional cross-breeding in *G. hirsutum*, which contributes 95% of the total cotton yield in the world [2]. Genetic engineering using plant resistance genes is becoming an alternative to improve cotton resistance to *V. dahliae*.

Cyclophilins (CYPs), possessing peptidyl prolyl *cis/trans* isomerase (PPIase) activity, are classified in the immunophilin family of proteins [3]. CYPs play important roles in various biological processes, including transcription regulation [4], protein folding [5], signal transduction [6] and ROS (reactive oxygen species) regulation [7]. With the availability of whole genome sequencing, the identification and characterization of plant CYPs are carried out mainly in *Arabidopsis thaliana* (29 AtCYPs) [8], *Oryza sativa* (27 OsCYPs) [9] and *Glycine max* (62 GmCYPs) [10]. The majority of studies reveal the involvement of plant CYPs mostly in different types of abiotic stress. For example, *Arabidopsis* CyPs showed evidence of response to wounding [11]. Rice *OsCYP19-4* showed over 10-fold up-regulation in response to cold. Overexpressing of *OsCYP19-4* could enhance rice plants cold-resistance with significantly increased tiller and spike numbers, and consequently enhanced grain weight [12]. Transgenic plants overexpressing *OsCYP21-4* exhibited increased tolerance to salinity and hydrogen peroxide treatment [13]. Ectopic expression of pigeon pea (*Cajanus cajan* L.) *CYP*, *CcCYP*, in *Arabidopsis* exhibited high-level tolerance against drought, salinity and extreme temperatures [14]. Against biotic stress, especially against pathogen infection, only several plant CYPs have been studied in plant-pathogen system. Pepper cyclophilin (*CACYP1*) gene expression increased in response to *Xanthomonas campestris* pv*. vesicatoria* and *Colletotrichum gloeosporioides* [15]. Fungal infection with *Fusariumsolani* f. sp*eumartii* increased the level of *Solanum tuberosusm* CyP gene *StCyP* mRNA in tubers [16]. The expression of *V. vinifera VviCyP* was highly induced by *Plasmopara viticola* [17]. In cotton, a cyclophilin-like gene *GhCyp1* was cloned from *G. hirsutum*. Overexpression of *GhCyp1* in transgenic tobacco plants conferred higher tolerance to salt stress and *Pseudomonas syringae* pv. *tabaci* infection compared with control plants [18].

In 2015, the genome of *G. hirsutum* L. acc. Texas Marker-1 (TM-1) was sequenced, more than 70, 000 protein-coding genes were predicted (NAU version 1.1) [19, 20]. Recently, an improved de novo–assembled genome for *G. hirsutum* L. acc. TM-1 were generated (NAU version 2.1) [21]. The genome-sequencing project facilitates the survey of all CYP genes in cotton*.* In the present study, the CYP gene family members in *G. hirsutum* and their expression patterns under various abiotic stresses and on *V. dahliae* infection were systematically investigated. Furthermore, the function of GhCYP-3 was analyzed to reveal its role in cotton resistance to *V. dahliae* infection. Our study will enlighten the novel insights into the function of CYP genes in plant against multivariate stress responses in the future and provide more candidate genes for resistance breeding in cotton.

## Results

### Up to 79 CYPs were identified in the genome of *G. hirsutum* TM-1

**Fig. 1:**
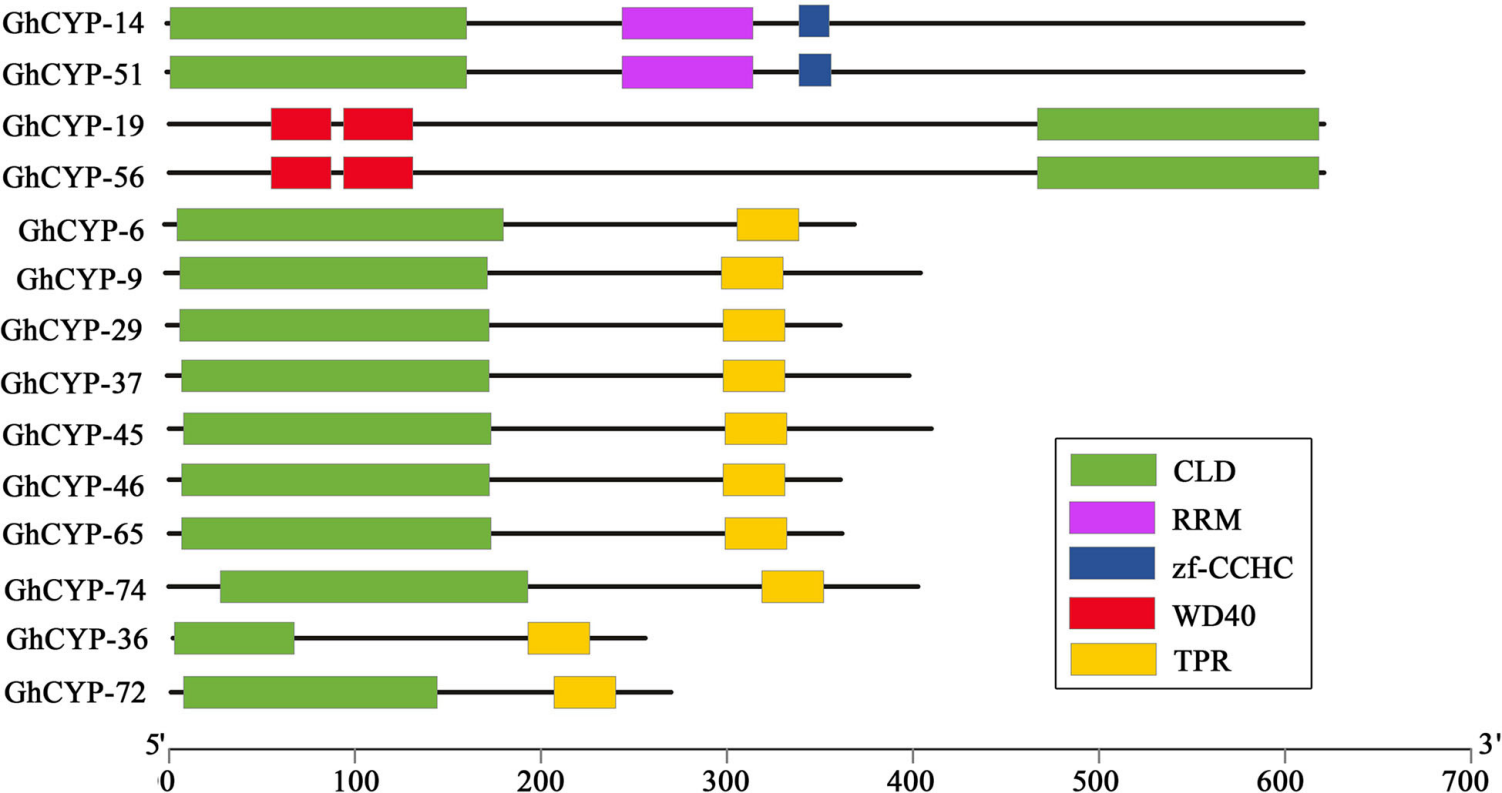
Schematic representation of multi-domain GhCYPs. CLD, cyclophilin-like-domain; RRM, RNA recognition motif; zf-CCHC, CCHC-type zinc finger protein; WD, tryptophan-aspartate repeat; TPR, tetratricopeptide repeat

A local BLASTP search was performed with the *Arabidopsis* CYP proteins as query, which resulted in 79 CYP candidates from *G. hirsutum* NAU version 1.1, 74 CYPs from *G. hirsutum* JGI version 1.0 and 78 CYPs from *G. hirsutum* NAU version 2.1 (Table 1). These candidates were submitted to Pfam to confirm the existence of cyclophilin type PPIase domain (CLD, PF00160) and named GhCYP-1 to GhCYP-79. The characteristics of the individual CYP, including CDS length, protein length, molecular weight, and isoelectric point (pI) were presented in Table 1. The protein length varied from 69 amino acid (aa) residues (GhCYP-70) to 801 aa (GhCYP-55). The molecular weight ranged from 7.5 kDa (GhCYP-70) to 90.5 kDa (GhCYP-55), and the pI values ranged from 4.6 to 12.0. Most of the GhCYPs were expected to be in the cytoplasm. Also, some GhCYPs exhibited chloroplast, mitochondrial, nuclear and extracellular localization. Of the 79 GhCYPs, 65 only contained one CLD domain, but the remaining 14 GhCYPs contain additional domains, including tetratricopeptide-like repeats (TPR, PF00515, PF07719, PF13181, PF13414), Zinc finger (zf-CCHC, PF00098), RNA recognition motif (RRM, PF00076) and WD40 (PF00400) (Fig. 1).

**Table 1:** The list of the putative CYP genes identified in *G. hirsutum*

Gene Name	Gene ID (NAU1)	CDS (bp)	Protein (aa)	MW (kDa)	pI	Subcellular location	Gene ID (JGI/NAU2)	Identity (%) (JGI/NAU2)
GhCYP-1	Gh_A01G0027	675	224	24.5	7.3	Cyto	Gohir.A01G003000/GH_A01G0029	100/100
GhCYP-2	Gh_A01G0031	516	171	18.2	7.9	Cyto	Gohir.A01G003400/GH_A01G0034	99/99
GhCYP-3	Gh_A01G1361	522	173	18.2	8.5	Cyto	Gohir.A01G164200/GH_A01G1763	100/100
GhCYP-4	Gh_A01G1747	495	164	18.0	8.2	Cyto/Mito/Chlo	Gohir.A01G204600/GH_A01G2217	100/100
GhCYP-5	Gh_A02G0528	708	235	26.5	9.8	Mito	Gohir.A02G054200/GH_A02G0571	100/100
GhCYP-6	Gh_A02G1526	1116	371	41.9	6.6	Cyto	No found/GH_A02G1850	-/100
GhCYP-7	Gh_A03G0499	966	321	35.0	8.7	Chlo	Gohir.A03G057400/GH_A03G0699	100/100
GhCYP-8	Gh_A03G0865	648	216	24.5	6.1	Chlo	Gohir.A03G099700/GH_A03G1148	100/100
GhCYP-9	Gh_A03G1688	1221	406	45.9	8.8	Cyto	Gohir.A03G191300/GH_A03G2153	100/100
GhCYP-10	Gh_A04G1046	522	173	18.5	10.1	Cyto	No found/GH_A04G1474	-/100
GhCYP-11	Gh_A04G1047	522	173	18.3	8.2	Cyto	No found/GH_A04G1475	-/100
GhCYP-12	Gh_A05G0642	1026	341	37.6	8.6	Cyto	Gohir.A05G078500/GH_A05G0800	74/73
GhCYP-13	Gh_A05G3461	567	188	20.4	8.2	Cyto	No found/GH_A05G4196	-/100
GhCYP-14	Gh_A05G4019	1836	611	70.5	6.2	Nucl	Gohir.A05G003300/GH_A05G0027	100/100
GhCYP-15	Gh_A06G0418	870	289	31.9	6.9	Extra	Gohir.A06G049800/GH_A06G0538	100/100
GhCYP-16	Gh_A06G0767	1494	497	56.1	8.7	Nucl	Gohir.A06G085500/GH_A06G0934	100/100
GhCYP-17	Gh_A07G0324	2373	790	89.3	12.0	Nucl	Gohir.A07G038000/GH_A07G0439	100/99
GhCYP-18	Gh_A07G0325	2388	795	89.9	11.6	Nucl	Gohir.A07G038100/GH_A07G0440	93/93
GhCYP-19	Gh_A07G0986	1866	621	70.0	7.0	Cyto	Gohir.A07G108500/GH_A07G1190	100/100
GhCYP-20	Gh_A07G2012	1359	452	50.0	4.9	Chlo	Gohir.A07G219400/GH_A07G2484	100/99
GhCYP-21	Gh_A08G0354	711	236	26.9	9.2	Mito	Gohir.A08G040200/GH_A08G0443	100/100
GhCYP-22	Gh_A08G1077	855	284	31.0	8.2	Extra/PM/Chlo	Gohir.A08G122000/GH_A08G1462	88/97
GhCYP-23	Gh_A08G1194	483	160	17.4	8.5	Cyto	Gohir.A08G133100/GH_A08G1597	100/100
GhCYP-24	Gh_A08G1470	1032	343	38.0	5.4	Extra	Gohir.A08G162600/GH_A08G1886	100/100
GhCYP-25	Gh_A08G1670	1986	661	72.8	10.7	Nucl	Gohir.A08G187500/GH_A08G2136	94/94
GhCYP-26	Gh_A09G0254	1308	435	47.9	4.6	Cyto	Gohir.A09G026100/GH_A09G0300	100/100
GhCYP-27	Gh_A09G0853	624	207	22.2	9.4	Cyto/Mito	Gohir.A09G090200/GH_A09G1082	96/96
GhCYP-28	Gh_A09G1765	1032	343	37.9	6.0	Extra	Gohir.A09G197200/GH_A09G2134	100/100
GhCYP-29	Gh_A10G0832	1089	362	40.3	6.7	Cyto	Gohir.A10G092800/GH_A10G0930	97/96
GhCYP-30	Gh_A10G1682	576	191	20.7	8.2	Cyto	Gohir.A10G187900/GH_A10G2053	100/100
GhCYP-31	Gh_A10G1687	609	202	22.1	8.6	Cyto	Gohir.A10G188300/GH_A10G2060	90/89
GhCYP-32	Gh_A10G2121	666	221	23.9	9.4	Cyto	Gohir.A10G236300/GH_A10G2615	100/100
GhCYP-33	Gh_A11G0678	996	331	37.2	5.3	Extra	Gohir.A11G074800/GH_A11G0773	82/82
GhCYP-34	Gh_A11G0987	705	234	26.8	8.9	Mito/Nucl	Gohir.A11G108500/GH_A11G1131	100/100
GhCYP-35	Gh_A12G0709	1791	596	65.3	8.2	Mito/Nucl	Gohir.A12G077300/GH_A12G0891	100/100
GhCYP-36	Gh_A12G2539	765	254	28.6	5.9	Cyto	Gohir.A12G082500/GH_A12G0712	100/100
GhCYP-37	Gh_A13G0333	1200	399	44.6	6.4	Cyto	No found/GH_A13G0375	-/100
GhCYP-38	Gh_A13G0846	525	174	18.8	7.8	Cyto	Gohir.A13G103900/GH_A13G1226	100/100
GhCYP-39	Gh_D01G0026	675	224	24.6	7.3	Cyto	Gohir.D01G002600/GH_D01G0028	100/100
GhCYP-40	Gh_D01G0030	516	171	18.2	7.4	Cyto	Gohir.D01G003100/GH_D01G0033	99/99
GhCYP-41	Gh_D01G0206	780	259	28.1	10.3	Chlo	Gohir.D01G020500/GH_D01G0221	100/100
GhCYP-42	Gh_D01G1605	522	173	18.3	8.5	Cyto	Gohir.D01G156100/GH_D01G1877	100/100
GhCYP-43	Gh_D02G0593	705	234	26.3	9.8	Mito	Gohir.D02G059200/GH_D02G0586	100/100
GhCYP-44	Gh_D02G1247	750	249	28.4	8.1	Chlo	Gohir.D02G124600/GH_D02G1353	98/100
GhCYP-45	Gh_D02G2108	1233	410	46.4	8.6	Cyto	Gohir.D02G212200/GH_D02G2325	100/99
GhCYP-46	Gh_D03G0186	1086	361	40.6	5.5	Cyto	Gohir.D03G020200/GH_D03G0207	99/99
GhCYP-47	Gh_D03G1033	966	321	35.0	8.2	Chlo	Gohir.D03G108800/GH_D03G1247	100/100
GhCYP-48	Gh_D04G1620	522	173	18.5	9.6	Cyto	Gohir.D04G164700/GH_D04G1813	100/100
GhCYP-49	Gh_D04G1621	522	173	18.3	8.2	Cyto	Gohir.D04G164800/GH_D04G1814	100/100
GhCYP-50	Gh_D04G1937	570	189	20.5	8.2	Cyto	Gohir.D04G016300/GH_D04G0183	100/100
GhCYP-51	Gh_D05G0033	1836	611	70.6	6.0	Nucl	Gohir.D05G003700/GH_D05G0030	100/100
GhCYP-52	Gh_D06G0456	870	289	31.9	7.3	Extra	Gohir.D06G049100/GH_D06G0504	100/100
GhCYP-53	Gh_D06G2331	1491	496	55.9	8.1	Nucl	Gohir.D06G084800/GH_D06G0925	100/100
GhCYP-54	Gh_D07G0381	2367	788	89.4	12.0	Nucl	Gohir.D07G042100/GH_D07G0441	100/99
GhCYP-55	Gh_D07G0382	2406	801	90.5	11.5	Nucl	Gohir.D07G042200/GH_D07G0442	98/97
GhCYP-56	Gh_D07G1064	1866	621	70.0	7.1	Cyto	Gohir.D07G112300/GH_D07G1170	100/100
GhCYP-57	Gh_D07G2233	1359	452	49.8	4.9	Chlo	Gohir.D07G226300/GH_D07G2429	100/99
GhCYP-58	Gh_D08G0452	711	236	26.7	9.5	Mito/Nucl	Gohir.D08G050300/GH_D08G0461	100/100
GhCYP-59	Gh_D08G1359	768	255	27.5	8.4	Chlo	Gohir.D08G143400/GH_D08G1489	89/88
GhCYP-60	Gh_D08G1477	483	160	17.4	7.8	Cyto	Gohir.D08G154400/GH_D08G1612	99/99
GhCYP-61	Gh_D08G1766	1032	343	38.0	5.5	Extra	Gohir.D08G182600/GH_D08G1902	100/100
GhCYP-62	Gh_D08G2018	1965	654	72.1	11.3	Nucl	Gohir.D08G205800/GH_D08G2160	100/99
GhCYP-63	Gh_D09G0253	1311	436	48.1	4.7	Cyto	Gohir.D09G025500/GH_D09G0307	100/100
GhCYP-64	Gh_D09G1874	1032	343	37.8	5.5	Extra	Gohir.D09G191800/GH_D09G2069	100/100
GhCYP-65	Gh_D10G0925	1089	362	40.3	6.9	Cyto	Gohir.D10G095600/GH_D10G1032	100/100
GhCYP-66	Gh_D10G1953	576	191	20.6	8.2	Cyto	Gohir.D10G195800/GH_D10G2172	100/100
GhCYP-67	Gh_D10G2442	666	221	23.8	9.4	Cyto	Gohir.D10G248100/GH_D10G2719	100/100
GhCYP-68	Gh_D11G0793	996	331	37.2	5.5	Extra	Gohir.D11G079500/GH_D11G0807	100/100
GhCYP-69	Gh_D11G1133	705	234	26.7	8.9	Mito/Nucl	Gohir.D11G113000/GH_D11G1161	100/100
GhCYP-70	Gh_D12G0709	210	69	7.5	9.1	Cyto/Nucl	Gohir.D06G090500/GH_D06G0997	98/94
GhCYP-71	Gh_D12G0724	1791	596	65.4	8.2	Cyto/Nucl	Gohir.D12G075500/GH_D12G0928	100/100
GhCYP-72	Gh_D12G0852	810	269	30.5	6.5	Cyto	Gohir.D12G089100/No found	99/-
GhCYP-73	Gh_D12G2822	519	172	18.1	8.5	Cyto	Gohir.D12G033300/GH_D12G0323	100/100
GhCYP-74	Gh_D13G0372	1212	403	45.4	7.2	Cyto	Gohir.D13G036300/GH_D13G0367	100/100
GhCYP-75	Gh_D13G1093	525	174	18.8	7.4	Cyto	Gohir.D13G107100/GH_D13G1170	100/100
GhCYP-76	Gh_Sca004717G03	1377	458	49.9	6.8	Chlo	Gohir.D11G293200/GH_A11G3184	100/99
GhCYP-77	Gh_Sca004717G12	1377	458	49.9	5.9	Chlo	Gohir.D11G296300/GH_D11G3210	100/100
GhCYP-78	Gh_Sca004880G02	780	259	28.1	10.5	Chlo	Gohir.A01G022000/GH_A01G0230	100/99
GhCYP-79	Gh_Sca006066G02	513	170	18.6	9.4	Cyto/Nucl	Gohir.D01G194000/GH_D01G2301	100/100

### *Cis*-elements potentially related to hormonal signal for *GhCYPs*

**Fig. 2:**
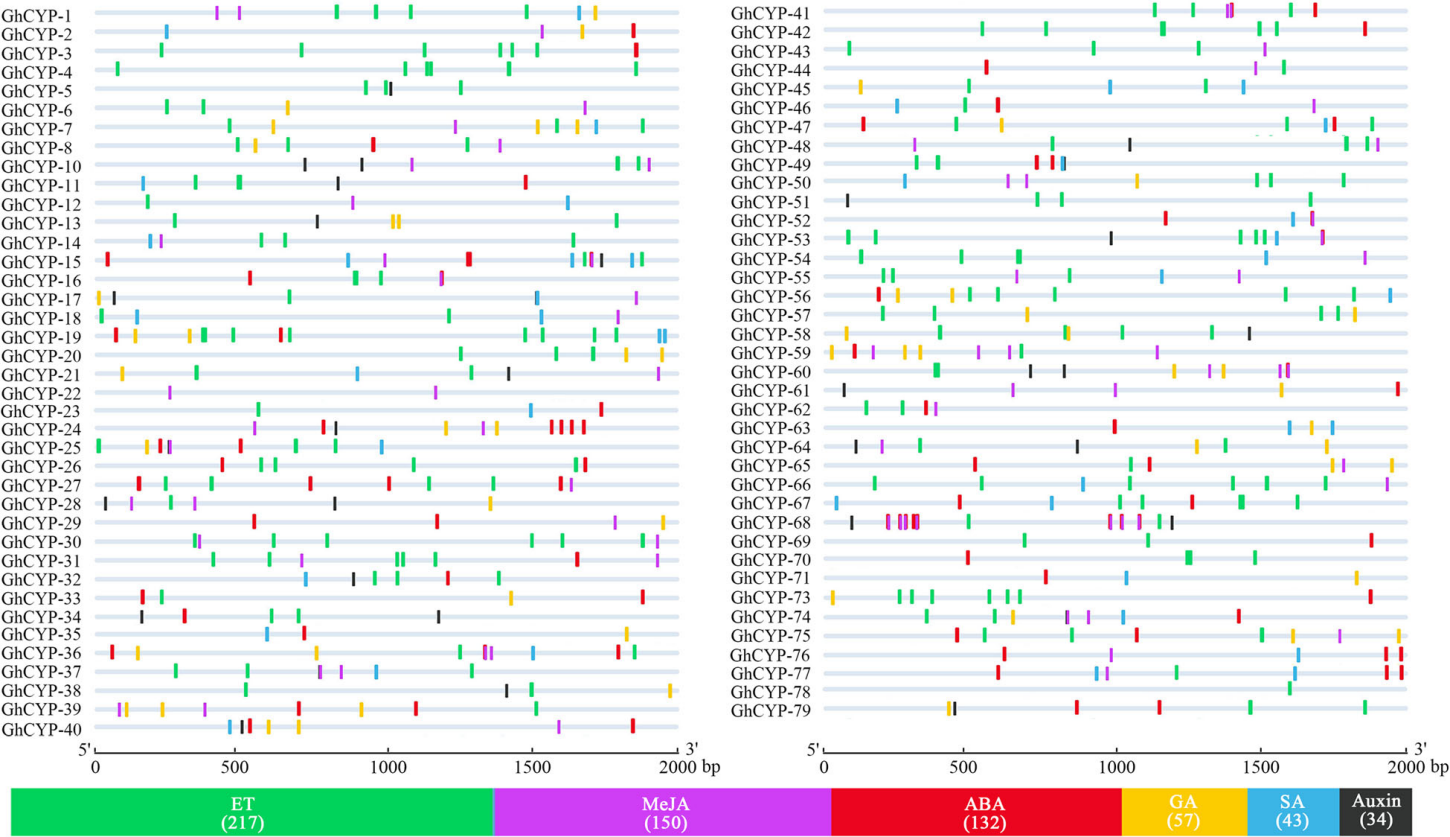
Potential *cis*-elements in a 2 kb 5′ flanking region upstream from the start codon of each *GhCYP* involved in phytohormone signaling. Potential *cis*-elements related to hormonal signal for *GhCYPs* are shown as colored bar at the bottom. The total number of *cis*-elements related to ET, MeJA, ABA, GA, SA and Auxin was shown in brackets.

Here we surveyed the presence of *cis*-elements potentially related to the hormonal signal, in the -2 kb 5′ flanking region upstream to the start codon of these *GhCYP*s. In total six types of hormones related *cis*-elements in the promoters were predicted (Fig. 2). Of these *GhCYPs*, 66 *GhCYPs* had ethylene (ET) responsive element (ERE), 35 *GhCYPs* contained salicylic acid (SA) responsive element (TCA-element), 47 *GhCYPs* harbored abscisic acid (ABA) responsive element (ABRE), 38 *GhCYPs* possessed gibberellin (GA) responsive element (P-box; TATC-box), 47 *GhCYPs* contained methyl jasmonate (MeJA) responsive element (CGTCA-motif), and 26 possessed auxin responsive element (TGA-box; AuxRR-core). In total, 217 *cis*-elements related to ET, 150 *cis*-elements related to MeJA, 132 *cis*-elements related to ABA, 57 *cis*-elements related to GA, 43 *cis*-elements related to SA and 34 *cis*-elements related to auxin were identified in all *GhCYPs* (Fig. 2). The enrichment of hormone-responsive *cis*-elements in the upstream of these *GhCYP*s suggests that they are likely to be involved in plant responses to various hormone signal pathways.

### Expression patterns of *GhCYPs* under various abiotic stresses

**Fig. 3:**
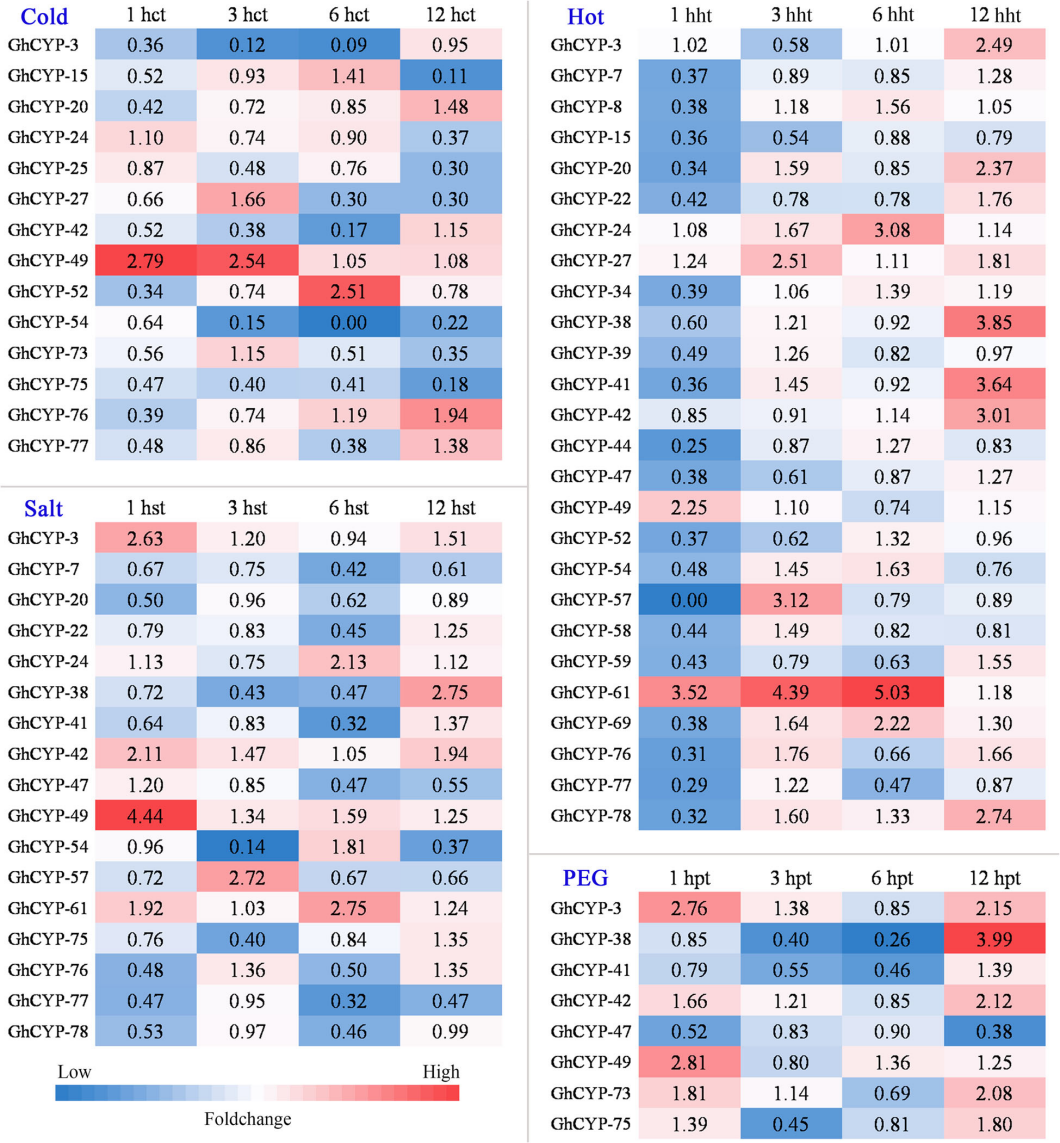
Expression profiles of *GhCYPs* in response to different abiotic stress. The fold change (FC) is the ratio of treatment FPKM to control FPKM. Red color indicates higher up-regulation expression. Blue color indicates lower down-regulation expression. The significantly differentially expressed genes were FC > 2 (up-regulated) or FC < 0.5 (down-regulated). hct, hours cold treatment; hht, hours hot treatment; hst, hours salt treatment; hpt, hours PEG treatment

Expression profiles of *GhCYPs* were examined in roots of cotton plants under four different abiotic stress conditions using high-throughput RNA-seq data (Additional file 2: Table S2). The transcripts with low Fragments Per Kilobase of exon per Million fragments mapped (FPKM) were probably false assembly. Therefore, in this study *GhCYPs* with FPKM >10 and present in at least two samples were identified as potentially expressed transcripts. We focused on the significantly differentially expressed genes (fold change [FC] > 2 or FC < 0.5) in the various stresses. *GhCYPs* that showed significant differential expression at least one treatment time were listed in Fig. 3. In the cold treatment, 14 *GhCYPs* were commonly down-regulated significantly apart from *GhCYP-49* at 1 hct (hours after cold treated) and 3 hct and *GhCYP-52* at 6 hct, which showed up-regulated significantly. At 1 hht (hours after hot treated), 22 of 26 *GhCYPs* are response to hot treatment. Of these, only the expression level of *GhCYP-49* and *GhCYP-61* was up-regulated significantly. At 3 hht, 6 hht and 12 hht, only 12 *GhCYPs* exhibited differential expression pattern.. Of these, up to 11 genes were up-regulated. Most *GhCYP* genes were found to be down-regulated under the condition of salt treatment apart from *GhCYP-3*, *GhCYP-24*, *GhCYP-38*, *GhCYP-42*, *GhCYP-49*, *GhCYP-57* and *GhCYP-61*. After PEG (polyethylene glycol) treatment, only 8 *GhCYPs* displayed differential expression pattern, of which, *GhCYP-3*, *GhCYP-42*, *GhCYP-49* and *GhCYP-73* were up-regulated, *GhCYP-41*, *GhCYP-47* and *GhCYP-75* were down-regulated. Only the expression of *GhCYP-38* showed be down-regulated at 3 hpt (hours after PEG treated) and 6 hpt, and then be up-regulated at 12 hpt. These expression patterns suggest that CYPs undertake multiple functions to help the cotton counter various complex environmental challenges.

### Expression profiles of *GhCYPs* under the stress of *V. dahliae*

**Fig. 4:**
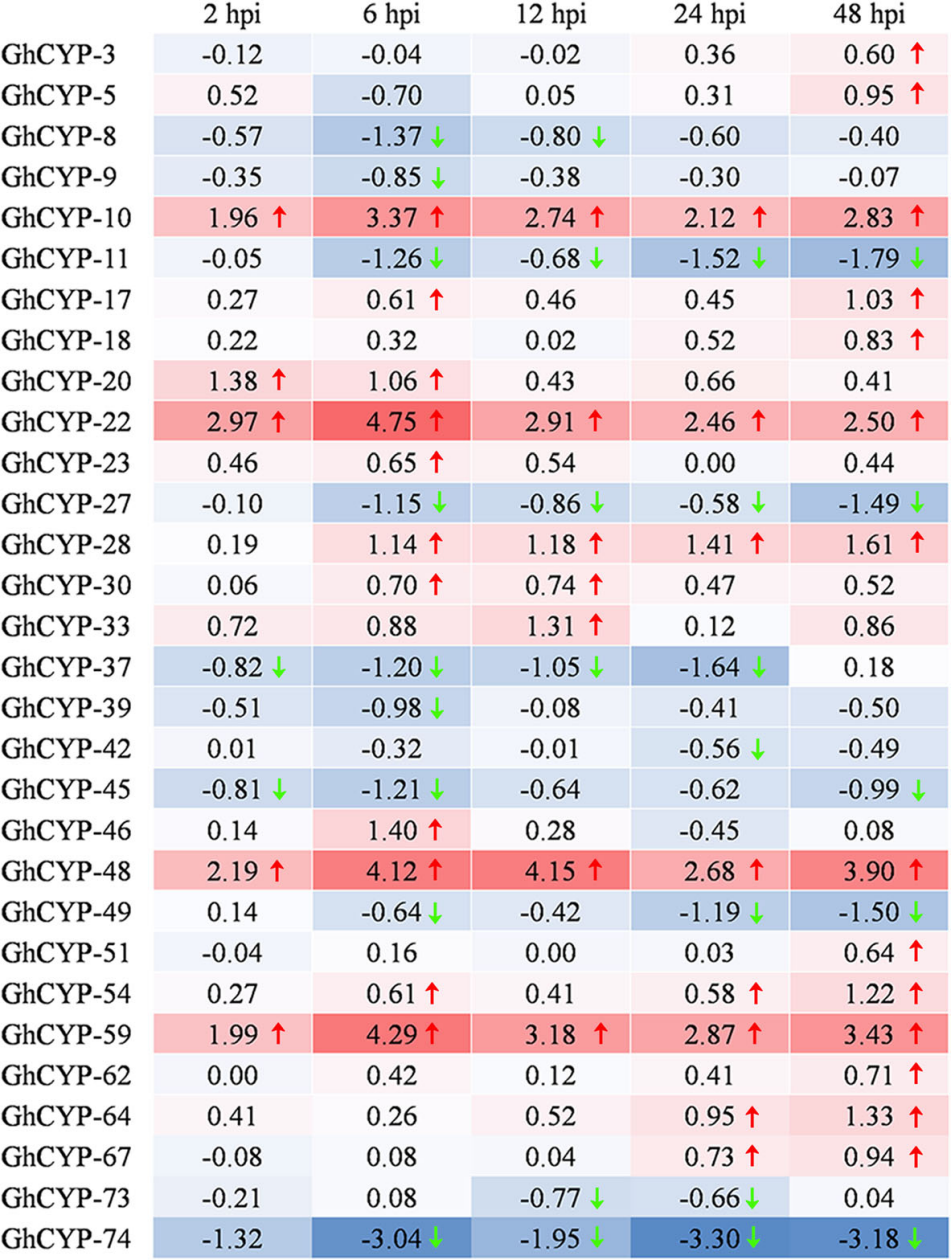
Expression profiles of *CYPs* from upland cotton inoculated with *V. dahliae*. Differential expression analysis was performed using the DESeq R package (1.10.1). The fold change (FC) is the ratio of treatment FPKM to control FPKM. Expression data are shown as log_2_^(FC)^. The resulting P-values were adjusted using the Benjamini and Hochberg’s approach for controlling the false discovery rate. Genes with an adjusted *P* < 0.05 according to DESeq were assigned as differentially expressed and marked with a red arrow (up-regulated) or green arrow (down-regulated). hpi, hours post inoculation

To gain a better understanding of the roles of *CYP* family genes in the cotton resistance against *V. dahliae*, the expression profiles were obtained by RNA-Seq. A total of 30 *GhCYP* genes showed differential expression at least one time point (hpi, hours post inoculation), including 19 upregulated genes and 11 downregulated genes (Fig. 4). Notably, levels of *GhCYP-10*, *GhCYP-22*, *GhCYP-48* and *GhCYP-59* were up-regulated at all five treatment time points. Additionally, the expression of *GhCYP-11*, *GhCYP-27*, *GhCYP-37*, *GhCYP-45* and *GhCYP-74* was down-regulated in most time points. These results revealed that *GhCYP*s were associated with the interaction between cotton and *V. dahliae.*

### GhCYP-3 contains conserved amino acid residues and has PPIase activity

**Fig. 5:**
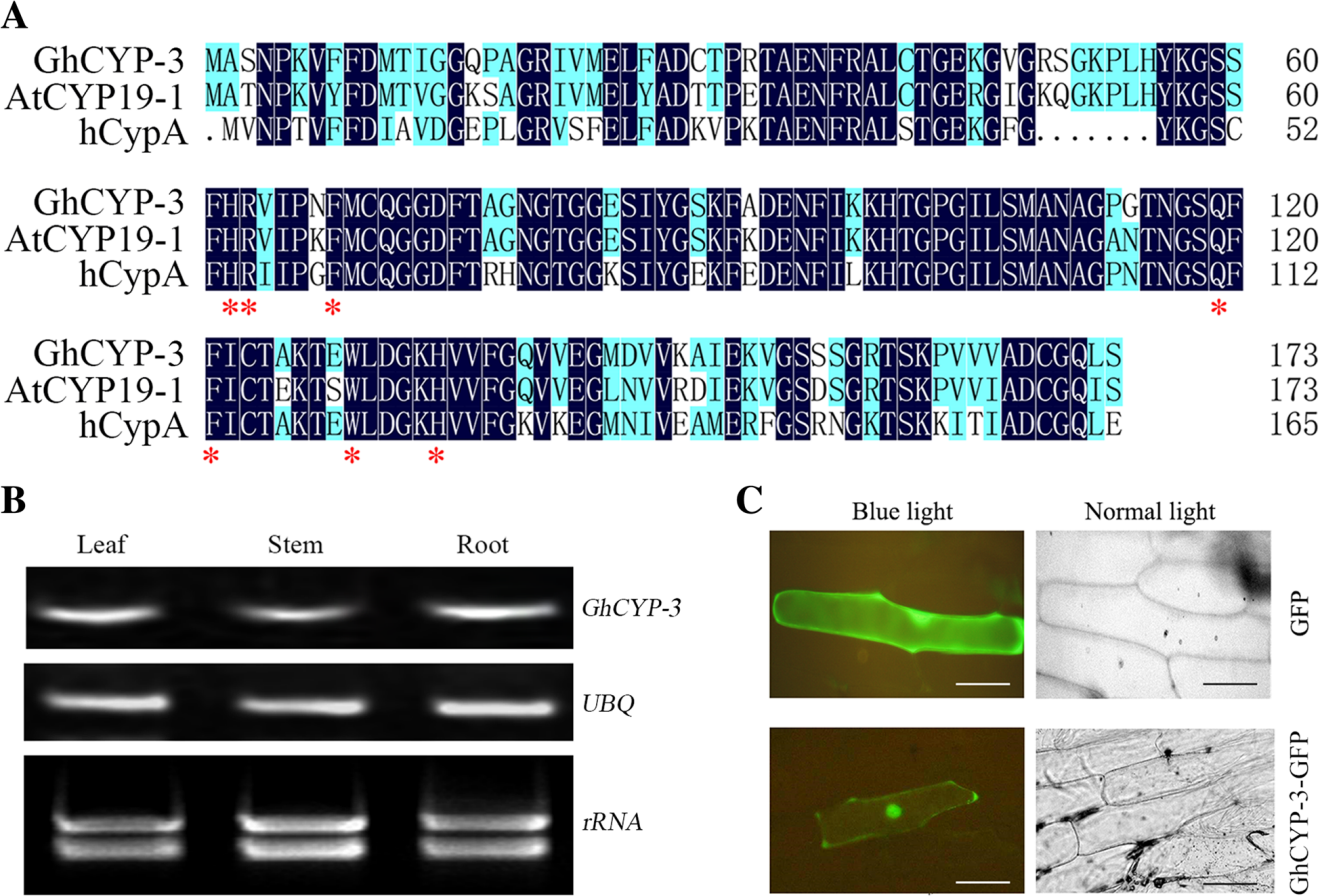
Characterization of GhCYP-3. **a**, Multiple sequence alignment of deduced amino acid sequence of GhCYP-3 with CYPs from *A. thaliana* (AtCYP19-1, At2g16600) and human (hCypA, NP_066953). Degree of homology for amino acid residues is indicated by color. Numbers on right side of sequences indicate the position of residues. Seven conserved active sites were marked with asterisks. **b**, Tissue-specific expression of *GhCYP-3*, based on semi-quantitative RT-PCR. **c**, Subcellular localization of GhCYP-3-GFP fused protein in onion epidermal cells. Scale bars indicate 50 μm

GhCYP-3, cloned from *G. hirsutum* cv. JM20, contains a single cyclophilin domain, 173 amino acid residues with a calculated molecular mass of 18.2 kDa and a pI of 8.34. Alignment with previously characterized *Arabidopsis* and human CYP (AtCYP19-1 and hCypA) revealed that GhCYP-3 contains seven conserved amino acid residues that critically affect PPIase activity (Fig. 5a). *GhCYP-3* was found to be expressed in root, stem and leaf of unchallenged cotton plants with *V. dalihae* (Fig. 5b). Translation fusion of GhCYP-3 with GFP was constructed under the control of 35S promoter, and then transiently expressed in onion epidermal cells. Fluorescent imaging of the GhCYP-3-GFP bombarded onion epidermal cells showed both cytoplasm and nuclear localization (Fig. 5c).

**Fig. 6:**
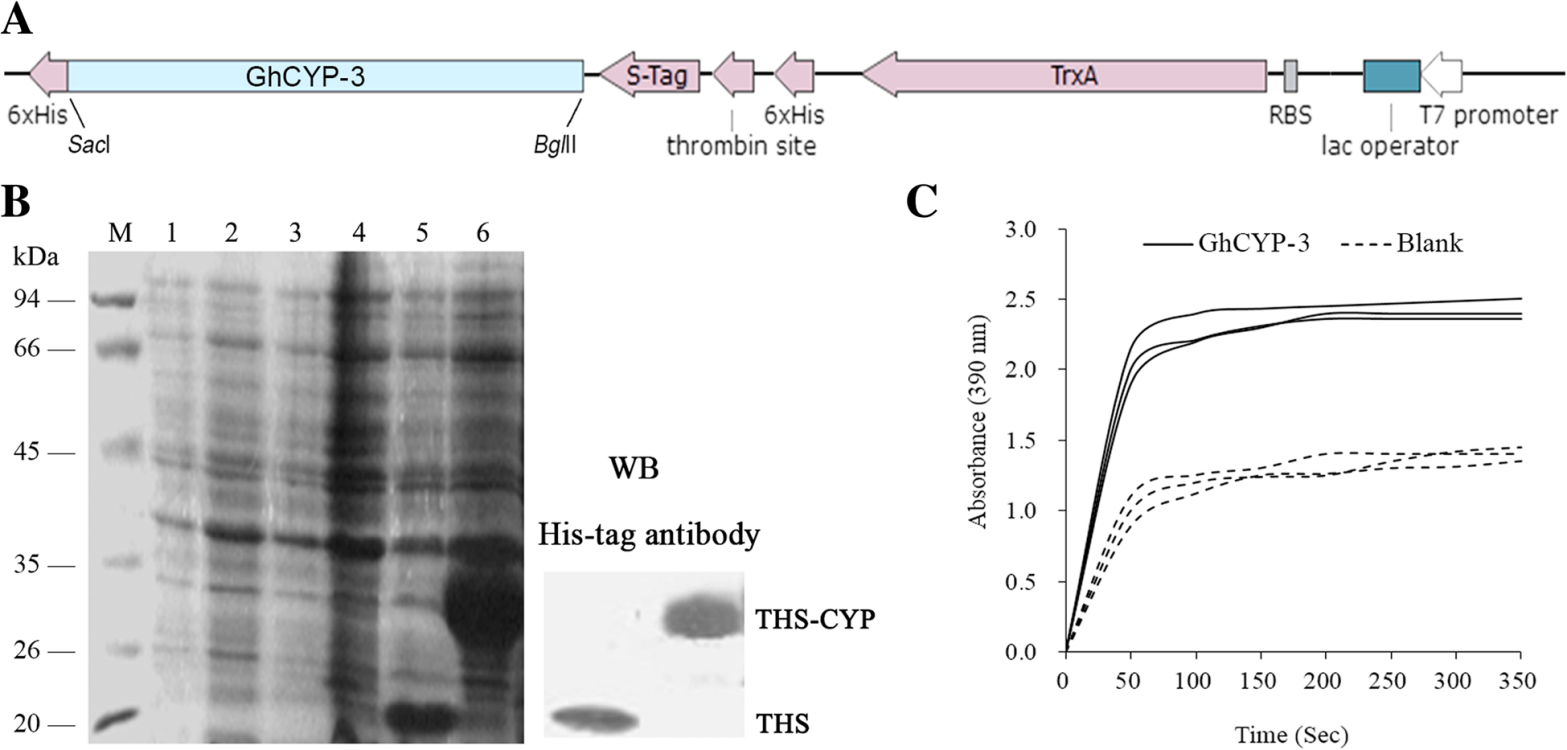
PPIase activity assays of recombinant GhCYP-3 in *vitro.* **a**, *GhCYP-3* with *Sac*I and *Bgl*II sites was amplified by PCR and cloned into the prokaryotic expression vector pET-32a. **b**, Expression and purification of recombinant GhCYP-3 in *Escherichia coli*. Recombinant GhCYP-3 expression was induced with 1 mM IPTG for 3 h. The resulting proteins were separated by 10% SDS-PAGE and analyzed by western blot using His antibody. 34.6 kDa Recombinant TrxA-6×His-S-tag-GhCYP-3 (THS-CYP) protein (arrows) was purified on nickel-NTA agarose columns. M, marker; Lane 1-2, empty vector pET-32a without IPTG; Lane 3-4, pET-32a- GhCYP-3 without IPTG; Lane 5, empty vector pET-32a with IPTG; Lane 6, pET-32a- GhCYP-3 with IPTG. **c**, A protease-coupled assay was used to measure PPIase activity of recombinant GhCYP-3. The prolyl cis-trans isomerization of the tetrapeptide substrate (Suc-Ala-Phe-Pro-Phe-2,4-difluoroanilide) was reflected by an increase in absorbance at 390 nm. The curves represent isomerization of the Suc-AFPF-pNA substrate over the course of 350 s in the absence of GhCYP-3 (Blank) and in the presence of 200 nM recombinant GhCYP-3 protein. Values represent the mean of three biological replicates

The GhCYP-3 ORF was cloned into pET-32a at *Sac*I and *Bgl*II sites, thus the recombinant plasmid was constructed (Fig. 6a). SDS-PAGE and western blot analysis revealed that the fusion TrxA-6×His-S-tag-6×His (THS, 20.4 kDa; empty vector as control) and TrxA-6×His-S-tag-GhCYP3 (THS-CYP, 34.6 kDa) proteins were highly expressed in the *E. coli* BL21(DE3) at 37°C with 1 mM IPTG for 4 h (Fig. 6b). The recombinant THS-CYP protein was purificated using the nickel-affinity. Further, we measured the PPIase activity of the recombinant protein by a coupled assay using synthetic peptide synthetic peptide succinyl- Ala-Ala-Pro-Phe-p-nitroanilide. The isomerization of the peptide substrate was observed in the presence of recombinant GhCYP-3, showing OD values higher than that of the blank, a negative control (Fig. 6c). These results indicated that GhCYP-3 has PPIase activity in *vitro*.

### GhCYP-3 expression is upregulated in response to *V. dahliae* invasion

**Fig. 7:**
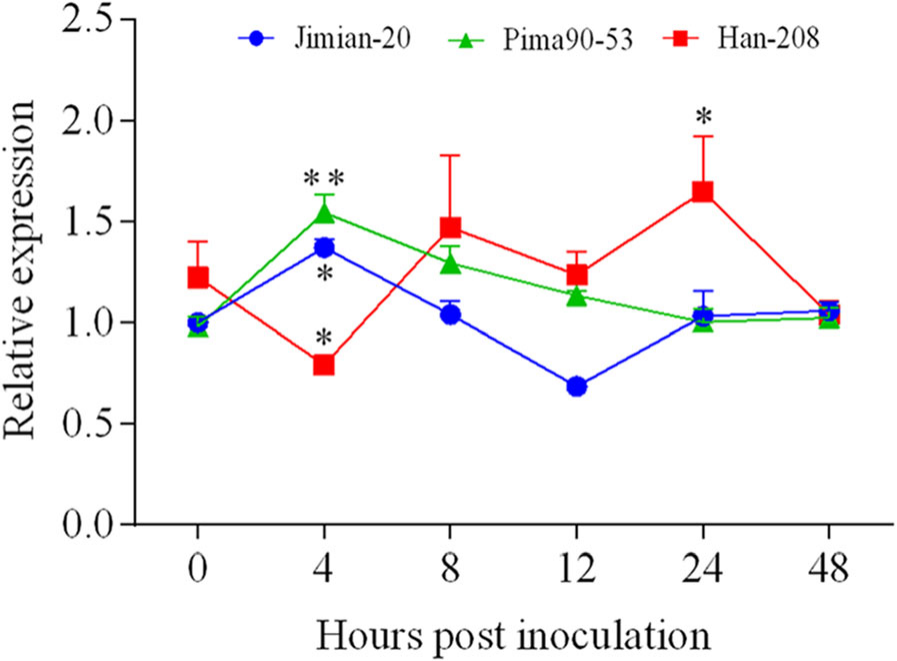
Expression patterns of *GhCYP-3* in three cotton cultivars with different degrees of resistance to *V. dahliae* Linxi2-1. The expression level of *GhCYP-3* in roots was normalized to *Ct* value for *GhUBQ14*. Data represent mean ± SE of three determinations. Significant differences at *p* = 0.05 (*) and *p* = 0.001 (**), based on Dunnett’s multiple comparison tests

To analyze the expression pattern of GhCYP-3 in three cotton cultivars with different degrees of resistance to *V. dahliae*, the roots of two-week-old cotton seedlings were sampled at 0, 4, 8, 12, 24 and 48 hpi. The qRT-PCR results revealed that the transcriptional levels of GhCYP-3, compared with 0 hpi, were significantly upregulated in two-resistant cultivars Pima90-53 and JM-20 at 4 hpi. However, at the same inoculation time, *GhCYP-3* in susceptible Han-208 was strongly downregulated and reached the highest expression level until 24 hpi (Fig 7). These results suggested that *GhCYP-3* is upregulated and earlier involved in the cotton interaction with *V. dahliae* in resistant than in susceptible cultivars*.*

### Overexpression of *GhCYP-3* in *Arabidopsis* improves plant Verticillium wilt resistance

**Fig. 8:**
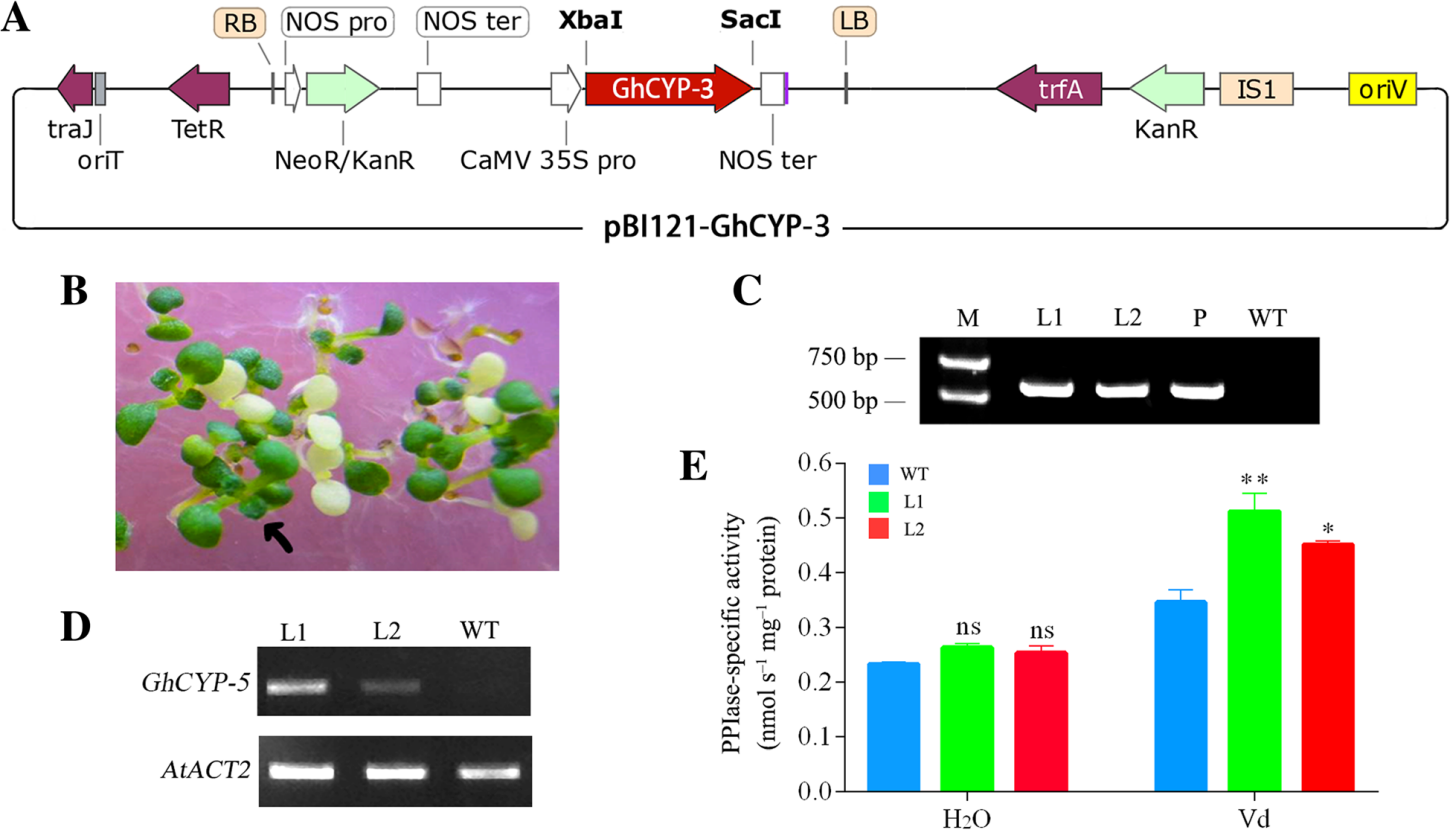
Genetic transformations of *Arabidopsis* with *GhCYP-3* and identification of transgenic lines. **a**, Schematic representation of pBI121-GhCYP-3 vector. **b**, Kanamycin resistance screening of positive seedlings; **c**, PCR analysis of genomic DNA from Kanamycin resistant lines. **d**, Semi-qPCR confirmed expression of *GhCYP-3* in transgenic lines. *AtACT2* (AT3G18780) was used as the internal standard. **e**, PPIase activity in wild-type (WT) and two transgenic lines. Data represent mean ± SE (n> 20) from three independent experiments. Asterisks indicate significant differences between WT and transgenic lines (Sidak's multiple comparisons test; ns= no significant, *P<0.05, **P<0.01). M, marker; L1 and L2, transgenic *Arabidopsis* lines; P, plasmid of pBI121-GhCYP-3 as positive control

To further evaluate whether the *GhCYP-3* functions in plant resistance to Verticillium wilt, we designed primers to amplify the ORF of *GhCYP-3* into the plant expression vector pBI121 (Fig. 8a), which will make *GhCYP-3* overexpression in transgenic *Arabidopsis* plants. By selection on kanamycin-containing medium (Fig. 8b), ten potential T_1_ transgenic lines were generated. After PCR-verification of gene insertion (Fig. 8c), two lines of the T_3_ generation displaying *GhCYP-3* overexpression (Fig. 8d) were selected for analysis of PPIase activity and Verticillium wilt resistance.

Under unstressed conditions (treated with H_2_O), no significant PPIase activity was enhanced in two GhCYP3-expressing transgenic lines compared to the WT (wild type). However, these two transgenic lines, infected with *V. dahliae* for 3 d, showed increased PPIase activity of 0.51±0.03 (L1) and 0.45±0.03 nmol·s^-1^·mg^-1^ protein (L2) compared to 0.35±0.03 nmol·s^-1^·mg^-1^ protein observed in the WT (Fig. 8e).

**Fig. 9:**
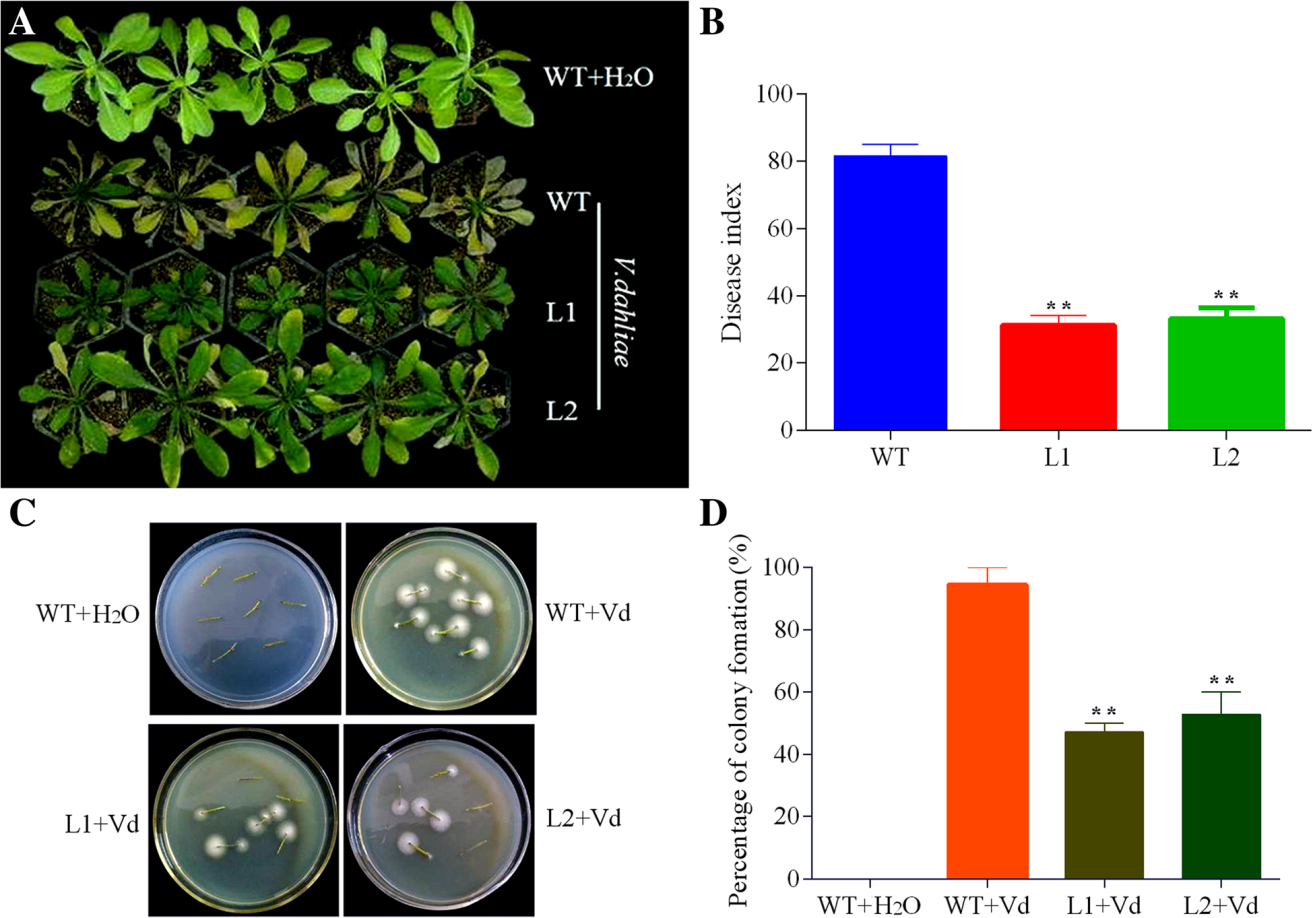
Enhanced disease tolerance of *Arabidopsis* plants overexpressing GhCYP-3. **a**, Phenotype comparison of wild-type (WT) and T3 independent GhCYP-3 transgenic plants (L1 and L2) inoculated with *V. dahliae* for 15 days. **b**, Disease indices of the WT and transgenic plants. Error bars indicate the SE (n>30) of three biological replicates. Asterisks indicate statistically significant differences as determined by Dunnett's multiple comparisons test (**P < 0.01). **c**, 10-dpi stem sections were plated on PDA medium. Colonies were formed after incubating 7 days at 25 °C. Vd, *V. dahliae.* **d**, Percentage of colony formation. Error bars indicate the SE (n = 18) of three biological replicates. Asterisks indicate statistically significant differences as determined by Dunnett's multiple comparisons test (**P < 0.01)

In response to *V. dahlia*e infection, two independent overexpressing lines displayed less wilting and smaller degree of leaf etiolation in comparison with WT plants at 15 dpi (Fig. 9a). Disease evaluation further indicated that the disease indices of transgenic lines were significantly lower than that of the WT (Fig. 9b). Furthermore, we examined the colonization in stems of infected WT and transgenic plants by isolation and cultivation of *V. dahliae* on PDA (potato dextrose agar) for 8 days. As a result, less fungal colonies came out from transgenic plants comparing to WT (Fig. 9c and d). These fungi recovery assay indicated that GhCYP-3 has a specialized effect of growth inhibition on *V. dahliae*.

### GhCYP-3 exhibited obvious inhibitory effects on *V. dahliae*

**Fig. 10:**
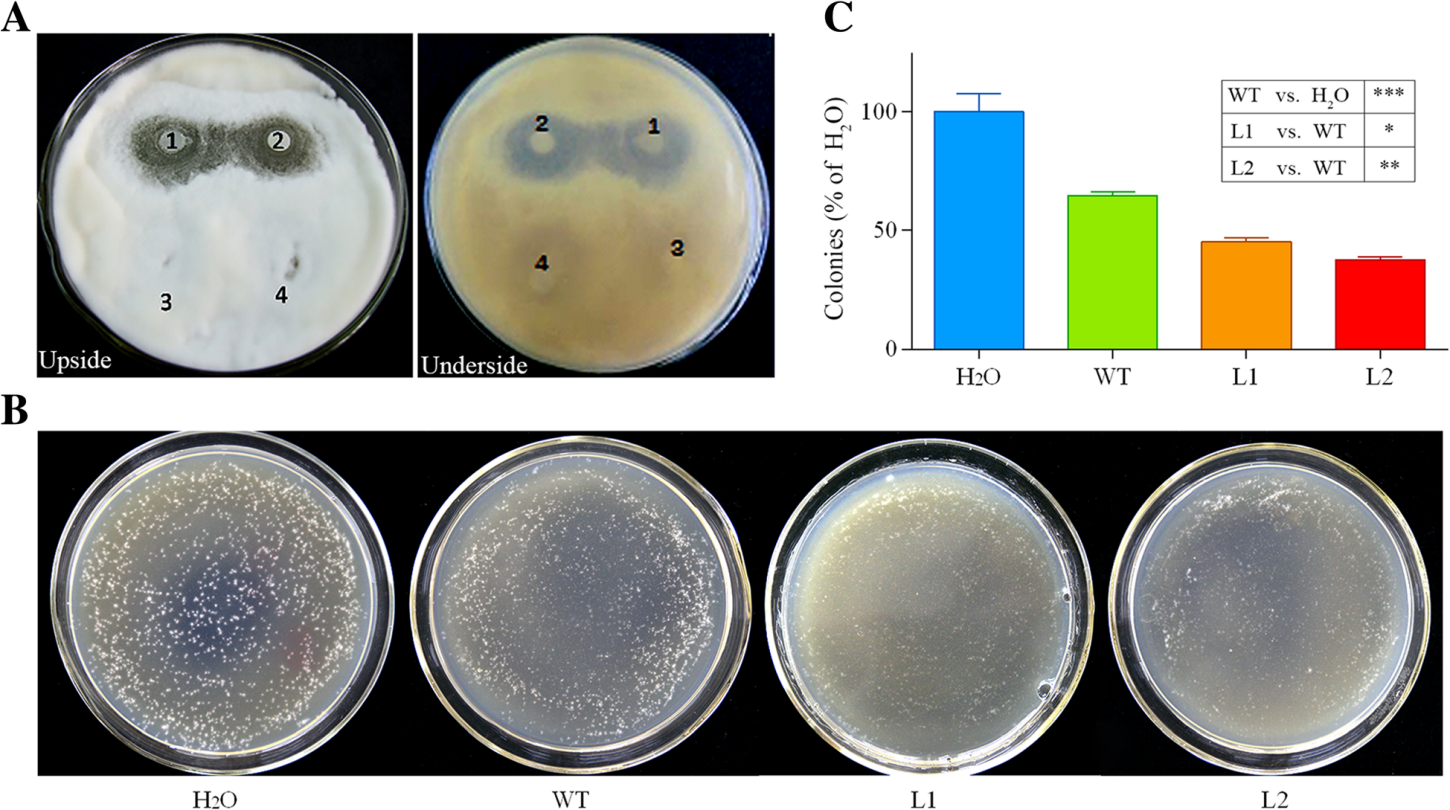
Inhibition of mycelial growth of *V. dahliae* by GhCYP-3. **a**, Inhibition of mycelial growth of *V. dahliae* by GhCYP-3 recombinant protein. The inhibition results were observed at 5 days after incubation. Clearing inhibition zones were formed around the disc containing recombinant GhCYP-3 (1 & 2). The disc containing water was as control (3 & 4). **b** and **c**, Extracts from transgenic plants significantly reducing the number of *V. dahliae* colonies. *V. dahliae* spores were inoculated with plant extracts and then be spread on PDA plates. After 48 h, the number of fungal colonies on plates contained transgenes extracts was drastically less than the H_2_O and WT. Data represent mean ± SE of three biological replicates. Asterisks indicate statistically significant differences as determined by Dunnett's multiple comparisons test (*P < 0.05, **P < 0.01, ***P < 0.001)

We further assessed the inhibitory effects of GhCYP-3 on *V. dahliae*. As showed in Fig. 10a, distinct inhibition zones formed around the disc containing recombinant GhCYP-3. Additionally, *V. dahliae* spores were inoculated with plant extracts from WT and transgenic lines and then be spread on PDA plates. After 48 h, extracts from all the plants significantly reduced the number of colonies compared to the H_2_O (as control). However, the number of fungal colonies on plates contain transgenes extracts was significantly less than the WT (Fig. 10b and c). These results indicated that GhCYP-3 can efficiently inhibit conidia germinating and hyphae growth of *V. dahliae* in *vitro*.

## Discussion

CYP genes family has been systematically analyzed in several plants, such as *Arabidopsis* [8], rice [22] and soybean [10]. Recently, 78 CYP genes were also identified in *G. hirsutum* L. acc. TM-1 from Illumina paired-end genomic sequencing (NAU version 1.1) by Chen et al. [23]. Now, NAU version 2.1, highly accurate reference grade genome assemblies and annotations for *G. hirsutum*, was generated [21]. A fairly large number of gaps and erroneous assemblies were successfully filled and corrected in new NAU version 2.1 [21]. Therefore, in the present study we identified a total of 79 CYP genes by integrating NAU version 1.1, JGI database and NAU version 2.1 (Table 1). Compared to the previous report from Chen et al., the supplementary three CYPs (GhCYP-49, GhCYP-60 and GhCYP-70) were identified in this study. Besides, two CYPs (Gh_Sca140771G01 and Gh_A12G1281), undiscoverable in JGI database and NAU version 2.1, were removed. It thus made the candidate CYP genes in *G. hirsutum* more reliable.

All 79 CYPs in *G. hirsutum* have conservative PPIase domain (CLD). Of which, 14 GhCYPs are multi-domain proteins (Fig. 1). In addition to the CLD, GhCYP-14 and GhCYP-51 contain RRM and zf-CCHC. The RRM domain was found in proteins involved in RNA processing where it mediates binding to various RNAs to execute both housekeeping functions and regulatory mechanisms [24]. Proteins that contain zinc fingers typically interact with DNA and RNA, and serve primarily to alter the binding specificity of a particular protein. In addition, GhCYP-14 and GhCYP-51 were predicted to be localized in the nuclear (Table 1). Therefore, these two CYPs possibly mediate ribosomal association while the CLD catalyzes peptidyl prolyl *cis-trans* isomerization of nascent polypeptides. GhCYP-19 and GhCYP-56 contain WD40 domain that generally serve as a rigid scaffold for protein interactions [25]. Other 10 multi-domain GhCYPs contain TPR domain, which mainly act as interactive scaffolds in the formation of protein complexes and regulators of RNA metabolism involved in the immune response [26, 27].

Regulation of gene expression *via* specific cis-regulatory elements in the promoter regions has evolved as a major adaptive mechanism to respond to environmental stress in plants [28]. Phytohormones are critical to the regulation of plant development and defense [29]. Thus, the analysis of the putative *cis*-regulatory elements relating to hormone helps to advance our understanding of *GhCYPs* involving stress tolerance in cotton. Six hormones (ET, MeJA, ABA, GA, SA and Auxin) responsive regulatory elements were detected in the potential promoter regions of GhCYPs (Fig. 2), indicating that GhCYPs involve different hormone-mediated signaling pathways.

In 2010, we isolated 203 ESTs from a cDNA library using suppression subtractive hybridization (SSH) with a resistant upland cotton cultivar Jimian20 induced with *V. dahliae*. Of which, an EST encoded a partial polypeptide with homology to CYP [30]. This is the first report that CYP is involved in the interaction between cotton and phytopathogen*.* In 2011, *GhCyp1* was cloned from *G. hirsutum* cv. Zhongmian 35. Overexpression of *GhCyp1* in transgenic tobacco plants conferred higher tolerance to salt stress and *P. syringae* pv. *tabaci* infection compared with control plants [18]. Here the RNA-seq expression analysis of the cyclophilin gene families for *G. hirsutum* revealed that many of the genes potentially play important roles in various stress response (Figs. 3 and 4). So far as we know, almost all of the reported CYP genes were involved in plant stress reactions by their up-regulation expression, such as *OsCYP19-4* and *OsCYP21-4* from rice against cold [12] and salt [13], *CcCYP* from pigeon pea against drought, salinity and extreme temperatures [14], CyPs from *Arabidopsis* against wounding [11], and several cyclophilin genes in response to phytopathogen infection [15–17]. Therefore, significantly higher expression of CYP genes in cotton, such as GhCYP-49 and GhCYP-52 to cold, GhCYP-3, GhCYP-24, GhCYP-42, GhCYP-49 GhCYP-57 and GhCYP-61 to salt, GhCYP-3, GhCYP-24, GhCYP-27, GhCYP-38, GhCYP-42, GhCYP-49 and GhCYP-61 to hot, GhCYP-3, GhCYP-42, GhCYP-49 and GhCYP-73 to PEG, and GhCYP-3, GhCYP-5, GhCYP-10, GhCYP-17, GhCYP-18, GhCYP-20, GhCYP-22, GhCYP-23, GhCYP-28, GhCYP-30, GhCYP-33, GhCYP-46, GhCYP-48, GhCYP-51, GhCYP-54, GhCYP-59, GhCYP-62, GhCYP-64 and GhCYP-67 to *V. dahliae* suggests likely functional importance under these stress conditions. However, further functional studies are required to unravel the precise role of these candidates during cotton response to biotic and abiotic stress conditions.

GhCYP-3 contained the above-mentioned EST sequence related to *V. dahliae-*infection and showed 98% similarity in amino acid sequences with GhCyp1. Thus, GhCYP-3 potentially plays an important role in regulating cotton immune response. The results of expression (Fig. 7) and overexpression analysis (Fig. 9) further supposed that *GhCYP-3* was involved in the cotton defence to *V. dahliae*. Plant CYPs could locate in multiple cell organelles, such as ER [31], chloroplast [9], Golgi [13], cytoplasm and nucleus [32, 33]. Interestingly, the GhCYP3-GFP fusion protein was localized in the cell cytoplasm as well as the cell nucleus in onion epidermal cells (Fig. 5c). Nuclear localization was proposed to play a possible role in the regulation of gene expression [33]. GmCYP1 was as a “helper” that activates the enzymatic activity of a *Phytophthora sojae* RXLR effector Avr3b in a PPIase activity-dependent manner [34]. Furthermore, GmCYP1 was demonstrated to interact with the isoflavonoid regulators GmMYB176 and 14-3-3 protein, suggesting that it participates in isoflavonoids metabolism and plays role in defense [33]. AtCYP57 was proved to be involved in the *A. thaliana* response to *P. syringae* infection by influencing callose accumulation and *PAD4* (peptidyl arginine deiminase type 4) expression, which interacts with EDS1 (enhanced disease susceptibility 1) to provide basal immune response in plants. Nucleus location makes authors inferred that AtCYP57 could directly regulate the translation of defence genes [32]. Therefore, we inferred that GhCYP-3 play resistance function, may like AtCYP57 and GmCYP1, in the cell nucleus by directly interacting with some transcription factor to regulate the translation of defence genes, which lead to the production of antimicrobial metabolites.

Alternatively, GhCYP-3 was also located in the cytoplasm, indicating that it needs to play some extra roles, presumably including direct antifungal activity. Antifungal activities of CYPs have been reported from some plants including ginseng [35], Chinese cabbage [36], chickpea [37] and black-eyed pea [38]. In our study, recombinant GhCYP-3 displayed evident inhibitory effects on *V. dahliae* on the plate (Fig. 10a). In a state of nature, the concentration of CYP in plant is hardly so high in the plate. Otherwise, there was no definite evidence to suggest that CYP is involved in plant resistance to pathogens by direct antifungal activity in vivo at present. However, we do not rule it out, because the extracts from *GhCYP-3* transgenic *Arabidopsis* displayed significantly inhibit activity to conidia germinating and hyphae growth of *V. dahliae* (Fig. 10b). Many characterized antifungal proteins active on the fungal cell wall, plasma membrane and or intracellular targets [39]. For example, Buforin 2 from the stomach tissue of *Bufo bufo gargarizans* can translocate through the plasma membrane and exhibits antifungal activity upon interaction with fungal DNA and RNA [40]. During the infection process, *V. dahliae* forms penetration peg and specialized fungus-host interface to secret secretory effector proteins [41]. This is probably a chance for antifungal proteins including GhCYP-3 produced by host plant encountering and entering into the cytoplasm of *V. dahliae.* Additionally, we identified 10 putative CYPs (VdCYPs) in *V. dahliae* strain VdLs.17 genome with very high sequence similarity with GhCYP-3 (Additional file 3: Table S3). These mean that GhCYP-3 may replace VdCYPs and carry out the same biological function in the cytoplasm of *V. dahliae.* Thus, we inferred that the antifungal activity of GhCYP-3 has been shown to be due to the effect on the normal function of VdCYPs, which is essential for the development of *V. dahliae*. Nevertheless, further research is needed to confirm this bold deduction.

## Conclusions

This is the first systematic analysis of CYP family genes in cotton aiming to help clarifying the gene sequence characteristics and expression patterns. The putative cis-regulatory elements predication and expression divergence suggested that the *GhCYP* genes are involved in multiple phytohormone regulation pathways and responses to various abiotic stress and *V. dahliae* infection. These results will provide potential clues for the selection of candidate genes for further in-depth study on the functional characterization. Furthermore, GhCYP-3 showed both cytoplasmic and nuclear localization. Heterologous overexpression of *GhCYP-3* in *Arabidopsis* significantly improved Verticillium wilt resistance of the plants. Recombinant GhCYP-3 and the extracts from *GhCYP-3* transgenic *Arabidopsis* displayed significantly inhibit activity to *V. dahliae*. These results indicated that *GhCYP-3* was associated with the resistance of cotton to *V. dahliae* infection presumably through antifungal activity, and it will offer an important candidate gene for Verticillium wilt tolerance in cotton molecular breeding.

## Methods

### Identification and characterization of CYP family genes in *G. hirsutum*

Genome assemblies of *G. hirsutum* TM-1 from Nanjing Agricultural University (NAU version 1.1 and version 2.1) and JGI (version 1.0) were retrieved from the CottonFGD website (https://cottonfgd.org/). *Arabidopsis* CYPs, accessed from TAIR website (https://www.Arabidopsis.org/) were used as query to identify putative CYPs in *G. hirsutum* genomes by local BLAST using BioEdit software (Ibis Biosciences, Carlsbad, CA, USA). The identified GhCYPs were further verified through the Pfam database (http://pfam.xfam.org). The molecular weight (MW) and isoelectric point (pI) of each protein were calculated using ExPASy program (http://www.expasy.org/). The signal peptide was predicted with the program of SignalP 4.1. The amino acid sequences were aligned with DNAMAN software (Vers. 7; Lynnon Corporation, Quebec, Canada), using default parameters. CELLO v2.5 (http://cello.life.nctu.edu.tw/) was used for the subcellular localization prediction of GhCYPs. The putative *cis*-acting elements in the promoter regions were predicted using NAU version 2.1 database with the Plant CARE (http://bioinformatics.psb.ugent.be/webtools/plantcare/html/).

### Genome-wide expression analysis of *GhCYPs*

A genome-wide expression analysis of the cotton CYP genes in various abiotic stresses and *V. dahliae* infection was performed using high-through RNA sequence data, which was downloaded from NCBI databases (SRP044705) and extracted from our RNA-seq data [42]. Genes with FPKM ≥ 10 were used for further expression analysis.

### Plant materials and *V. dahliae* strains

The cotton seeds *G. hirsutum* cv. Ji Mian 20 (JM20), Han208, CCRI8 and *G. barbadense* cv. Pima90-53 were preserved at the North China Key Laboratory for Crop Germplasm Resources of Education Ministry, Hebei Agricultural University, Baoding, China. Cotton seedlings were grown in commercial sterilized soil at 28°C /25°C (day/night) temperatures with a 16-h-light/8-h-dark regime. *A. thaliana* was grown in pots containing vermiculite soil with temperature at 23°C day and 20°C night, under a 16/8 h photoperiod. *V. dahliae* strain Linxi2-1 was isolated from a symptomatic upland cotton plants growing in agricultural fields in Linxi county, Hebei Province, China [43]. These highly aggressive defoliating *V. dahliae* strains were maintained on PDA. The conidial suspension was prepared according to previous description [44] and adjusted to 10^7^ spores per milliliter and 10^6^ spores per milliliter with distilled water was used to the inoculation of cotton and *Arabidopsis*, respectively. The plant was infected with *V. dahliae* using soil drench method [44]. 10 mL of the conidial suspension was directly injected with a needle without piercing into the bottom of each pot. Seedlings received sterile water in the same manner were used as control.

### Gene cloning and subcellular localization

Total RNA was extracted from leaf tissues of JM20 with an RNA plant plus reagent (TIANGEN Biotech, China). First-strand cDNA was synthesized from an aliquot of 1 μg of total RNA with a PrimeScript™ RT Reagent Kit and gDNA Eraser (TaKaRa, China). *GhCYP-3* was amplified with primers CYP-F1 and CYP-R1 (Additional file 1: Table S1), designed based on the sequences of Gh_A01G1361 (*G. hirsutum* L. acc. TM-1) [20]. A *GhCYP-3*-GFP fusion construct under the control of the 35S promoter was generated by cloning the ORF into the *Sal*I and *Bam*HI sites of the binary vector pCamE. The vector expressing GFP alone served as control. Protein subcellular localization in onion (*Allium cepa*) epidermal cells was determined according to the protocol of Yang (2015) [44].

### qRT-PCR and semi-quantitative PCR

Total RNA and cDNA were prepared by the method described above. The qRT-PCR was performed on a CFX96 Real-Time PCR Detection System (Bio-Rad, Hercules, CA, USA) using the qPCR kit for SYBR Green (TaKaRa, China). qPCR conditions consisted of one cycle of 3 min at 95 °C, followed by 40 cycles of 15 s at 95 °C, 15 s at 58 °C, and 20 s at 72 °C. The cotton ubiquitin 14 (*UBQ14*) gene served as an internal standard [45]. Fold-changes in expression were calculated via the 2^*−ΔCt*^ method. Semi-quantitative RT-PCR was done with an Applied Biosystems® 2720 Thermal Cycler with *Arabidopsis AtACT2* (AT3G18780) as the internal standard [46]. The resulting products were resolved on a 1.5% agarose gel. All primers are listed in Additional file 1: Table S1 Three biological and three technical replicates were analyzed for all quantitative experiments.

### Generation and evaluation resistance of transgenic *Arabidopsis*

*GhCYP-3* was cloned into pBI121 vector at the *Xba*I and *Sac*I sites by PCR with primers CYP-*X* and CYP-*S* (Additional file 1: Table S1). The chimeric construct was introduced into *Agrobacterium tumefaciens* strain GV3101 for *Arabidopsis* transformation using the floral dip method [47]. Putative transformants were selected on MS (Murashige and Skoog) medium containing 50 mg L^-1^ kanamycin, and then be further verified by PCR for gene insertion and semi-quantitative RT-PCR for gene expression. Independent T_1_ transgenic lines were used to produce the T_3_ generations, which were randomly chosen as representative lines and subjected to analysis. Disease severity of *Arabidopsis* plants caused by *V. dahliae* was assessed according to symptoms manifested on the leaves. The disease index (DI) was calculated as previously described [48]. Fungal recovery assay was also used to evaluate the resistance of the plant through assessment of Verticillium colonization recovered from stem sections according to the method of Fradin (2009) [49]. In each treatment, 18 individual plants were used and all the experiments were repeated thrice.

### Purification and PPIase activity assay of recombinant protein

The *GhCYP-3* ORF was cloned into expression vector pET-32a (+) (Novagen, Darmstadt, Germany) with forward primer CYP-*Bg*-F and reverse primer CYP-*Sa*-R (Additional file 1: Table S1), which will introduce the *Bgl*II and *Sac*I site into the 5′ and 3′ end of the ORF, respectively. To induce the expression of GhCYP-3 protein with a His-tag in *E. coli* BL21(DE3) (TransGen Biotech, Beijing, China), a final concentration of 1.0 mmol·L^−1^ isopropyl-β-D-thiogalactopyranoside (IPTG) was added into the culture when the OD_600_ value reached 0.4–0.6, and the culture was allowed to continue growing for 4 – 6 h before harvesting. The proteins were separated on a SDS–PAGE gel and detected by western blot using anti His-Tag mouse monoclonal antibody (1:5000; CW Biotech, Beijing, China). The recombinant protein was purified using a 6×His-Tagged Protein Purification Kit (CW Biotech, Beijing, China). The PPIase activity of the recombinant protein was assayed *in vitro* using the tetrapeptide substrate Suc-AAPF-pNA (N-succinyl-Ala-Leu-Pro-Phe-p-nitroanilide; Sigma-Aldrich, Ontario, Canada) in a Shimadzu UV-2450 spectrophotometer (Shimadzu, Kyoto, Japan) as described by Yoon (2016) [12]. Three biological and two technical replicates were analyzed for all measurement.

### Assay of antifungal activity

The antifungal activity of the recombinant protein was tested against *V. dahliae* using filter paper disc diffusion method. *V. dahliae* spores were uniformly spread on the PDA medium plates and then cultured for 60 h at 28°C. Sterilized blank paper discs of 6 mm diameters, impregnated with tested protein, were placed on the surface of PDA medium previously spread with *V. dahliae*. The plates were inoculated at 25°C for 10 d. The antifungal activity of extracts from *Arabidopsis* plants transformed with *GhCYP-3* was performed as described previously [50] with the following modifications. Conidial suspension adjusted to a density of 10^5^ conidia ml^-1^, and germinated on PDA overnight at 25 °C prior to assay. Total homogenates (5 g) from ten *Arabidopsis* plants were prepared by directly grinding plant leaves into a fine powder in liquid nitrogen with no buffer added. Subsequently, extracts were collected by centrifugation at 10000 g for 10 min at 25 °C. Conidial suspensions (25 μl) were mixed with 225 μl of extract, and incubated for 1 h at 25°C. The mixture (50 μl) were spread onto PDA plates and incubated at 25 °C for 48 h and fungal colonies enumerated. All experiments were repeated three times independently.

### Statistical analysis

All experiments were performed at least three times for each determination. Statistical analysis was performed using GraphPad Prism® 6 software (Graph Pad, San Diego, CA, USA). Unless otherwise indicated, data were evaluated by using analysis of variance (ANOVA) followed by Dunnett's multiple comparisons test.

## Additional files

Additional file 1: **Table S1.** Primers list (DOCX 15 kb)

Additional file 2: **Table S2.** The transcripts of *GhCYPs* with FPKM in response to abiotic stress (XLSX 22 kb)

Additional file 3: **Table S3.** Putative CYPs identified in *V. dahliae* strain VdLs.17 (DOCX 13 kb)

## Data Availability

The data generated or analyzed during the current study are included in this published article and its supplemental data files and available from the corresponding author on reasonable request.

## Abbreviations

ABA: Abscisic acid
ABRE: Abscisic acid responsive element
CLD: Cyclophilin type PPIase domain
CYP: Cyclophilin
DI: Disease index
EDS1: Enhanced disease susceptibility 1
ERE: Ethylene responsive element
ET: Ethylene
FC: Fold change
FPKM: Fragments per kilobase of exon per million fragments mapped
GA: Gibberellin
hct: Hours after cold treated
hht: Hours after hot treated
hpi: Hours post inoculation
hpt: Hours after PEG treated
MeJA: Methyl jasmonate
MS: Murashige and Skoog
*PAD4*: Peptidyl arginine deiminase type 4
PDA: Potato dextrose agar
PEG: Polyethylene glycol
pI: Isoelectric point
PPIase: Peptidyl prolyl cis/trans isomerase
ROS: Reactive oxygen species
RRM: RNA recognition motif
SA: Salicylic acid
TPR: Tetratricopeptide-like repeats
WT: Wild type
