# Supplementary material for: Genome-wide identification of cyclophilin genes in Gossypium hirsutum and functional characterization of a CYP with antifungal activity against Verticillium dahliae
Source: BMC Plant Biol. 2019 Jun 21;19:272. doi: 10.1186/s12870-019-1848-1 (PMC6588949; doi:10.1186/s12870-019-1848-1)
Supplement: Supplementary file 1 — Table S1. Primers list (DOCX 15 kb) [file 12870_2019_1848_MOESM1_ESM.docx]

| **Primer name** | **Primer sequence (5' to 3')** | **Use and specification** |
| --- | --- | --- |
| CYP-F1  CYP-R1 | ATGGCCTCAAATCCCAAG  CTAAGAGAGCTGTCCGCAGTC | For cloning of *GhCYP-3* from upland cotton JM20 |
| CYP‐*X*  CYP‐*S* | TCGAGAATGGCCTCAAATCCCAAGGTCTTCT^a^  GAGCTCCTAAGAGAGCTGTCCGCAGTCAGC^b^ | For cloning of *GhCYP-3* into pBI121 vector |
| CYP‐*Sa*  CYP‐*Ba* | GTCGACATGGCCTCAAATCCCAAGGTCTTCT^e^  GGATCCCTAAGAGAGCTGTCCGCAGTCAGC^f^ | For cloning of *GhCYP-3* into pCamE vector |
| CYP-RT-F  CYP-RT-R | GCTGGTCGGATTGTGATGGC  GAAGAGGAGCCTTTGTAGTGGAG | For RT-qPCR analysis of *GhCYP-3* |
| GhUBQ14-F  GhUBQ14-R | CAACGCTCCATCTTGTCCTT  TAGTCGTCTTTCCCGTAAGC | As the internal standard |
| Actin-F  Actin-R | CTTGCACCAAGCAGCATGAA  CCGATCCAGACACTGTACTTCCTT | Housekeeping genes of *A. thaliana* (At3g18780) for Semi-RT-qPCR |
| CYP-F2  CYP-R2 | TTCTTTGACATGACTATCGGTGG  TAAGAGAGCTGTCCGCAGTCA | Positive selection of transgene *A. thaliana* by PCR |
| CYP‐*Bg*-F  CYP‐*Sa-*R | CCTAGATCTCACCATGGCCTCAAATCCCAAG^c^  CACGAGCTCCTAAGAGAGCTGTCCGCAGTC^d^ | For cloning of *GhCYP-3* into pET32a vector |
| Restriction sites are underlined.  ^a^ *Xba*I  ^b^ *Sac*I  *^c^ Bgl*II  *^d^ Sac*I  ^e^*Sal*I  ^f^*Bam*HI | | |

Table S1. Primers list
