# Supplementary material for: Genome-wide identification of cyclophilin genes in Gossypium hirsutum and functional characterization of a CYP with antifungal activity against Verticillium dahliae
Source: BMC Plant Biol. 2019 Jun 21;19:272. doi: 10.1186/s12870-019-1848-1 (PMC6588949; doi:10.1186/s12870-019-1848-1)
Supplement: Supplementary file 3 — Table S3. Putative CYPs identified in V. dahliae strain VdLs.17 (DOCX 13 kb) [file 12870_2019_1848_MOESM3_ESM.docx]

**Table S3** Putative CYPs identified in *Verticillium dahliae* strain VdLs.17

| Accession No. | Identity to GhCYP-3 (%) |
| --- | --- |
| VDAG_00095 | 63 |
| VDAG_01004 | 60 |
| VDAG_01417 | 60 |
| VDAG_06261 | 55 |
| VDAG_01916 | 48 |
| VDAG_04131 | 46 |
| VDAG_06633 | 46 |
| VDAG_04038 | 42 |
| VDAG_07025 | 34 |
| VDAG_02606 | 33 |
